# Does K-wire fixation improve outcomes in children with a Seymour fracture?

**DOI:** 10.1016/j.amsu.2022.104566

**Published:** 2022-09-09

**Authors:** Riki Houlden

**Affiliations:** East & North Hertfordshire NHS Trust, Stevenage, UK

## Abstract

**Introduction:**

The Seymour fracture is a juxta-epiphyseal fracture of the terminal phalanx of the finger. Sources vary on the recommended management, with some advocating treatment without K-wires to avoid metalwork-associated infection, and others suggesting that K-wire fixation is necessary due to the risks of fracture re-displacement.

**Methods:**

A best evidence topic in paediatric hand surgery was written according to a structured protocol. Searches were performed on December 28, 2021 in Cochrane library and PubMed.

**Results:**

69 papers were found using the reported search strategy, and eight papers representing the best evidence to answer this question are discussed.

**Discussion:**

The evidence on this subject is suboptimal as five of these studies were case-series that do not make direct comparisons between the question's intervention and control groups, and the other three were single-centre retrospective cohort studies with no randomisation.

**Conclusion:**

The best evidence topic concludes that K-wire fixation appears to be associated with a higher rate of physeal disturbance and lower rates of infection, fracture re-displacement, and flexion deformity.

## Highlights

• K-wire fixation appears to be associated with higher rates of physeal disturbance.
• And with lower rates of infection, fracture re-displacement, and flexion deformity.
• Most will have normal clinical and radiographic findings at long-term follow-up.

## 1 Introduction

The characteristics of the Seymour fracture and its management were first described in detail in 1966 [1]. It is a juxta-epiphyseal fracture of the terminal phalanx of the finger. The diagnosis should be considered in a child presenting with a mallet finger deformity and the base of the nail lying superficial to the proximal nail fold. The diagnosis can be confirmed with a lateral radiograph demonstrating volar angulation of the diaphysis on the epiphysis. Sources vary on the recommended management, with some advocating treatment without K-wires to avoid metalwork-associated infection [1], and others suggesting that K-wire fixation is necessary due to the risks of fracture re-displacement [2].

A best evidence topic was constructed according to a structured protocol as described in a previous publication in the IJS [3] to determine whether children with a Seymour fracture managed with K-wire fixation have improved clinical outcomes relative to those managed without K-wire fixation.

## 2 Clinical scenario

A 12-year-old boy presents to the emergency department with a painful and deformed right ring finger after jamming it in a door. The distal interphalangeal joint appears to be in slight flexion at rest, the nail plate appears longer than those of the other fingers with signs of bleeding from the nailbed, and plain films demonstrate a physeal fracture of the distal phalanx with volar angulation. The diagnosis of a Seymour fracture is made. A colleague comments that K-wire fixation is typically required but some elect to manage the fracture conservatively. You wonder what the effect of K-wire fixation is on patient outcomes.

## 3 Three-part question

In children with a Seymour fracture (patient), does K-wire fixation (intervention) compared with no K-wire fixation (comparison) influence clinical findings, radiographic findings, and complication rates (outcomes)?

## 4 Search strategy

Searches were all performed on December 28, 2021.

### 4.1 Cochrane library

Search [All Text]: ((seymour* AND fracture*) OR (fracture* AND distal AND phalan* AND (phys* OR epiphys* OR base*))) AND (K-wir* OR Kirschner OR wir* OR fixat* OR pin*).

### 4.2 PubMed

Search: ((seymour*[Title/Abstract] AND fracture*[Title/Abstract]) OR (fracture*[Title/Abstract] AND distal[Title/Abstract] AND phalan*[Title/Abstract] AND (phys*[Title/Abstract] OR epiphys*[Title/Abstract] OR base*[Title/Abstract]))) AND (K-wir*[Title/Abstract] OR Kirschner*[Title/Abstract] OR wir*[Title/Abstract] OR fixat*[Title/Abstract] OR pin*[Title/Abstract]). Filter applied: Humans.

In addition, the reference lists of the relevant papers were searched.

## 5 Search outcome

Search results: 69 references, seven relevant to the question. One further relevant reference identified as a secondary reference.

## 6 Results

The results of the eight papers are summarised in Table 1.

**Table 1:** Summary of search results relevant to question.

Author, date and country	Patient group	Study type and level of evidence	Outcomes	Key results	Comments
Seymour, 1966 [1], England	20 patients, time to follow-up not specifically disclosed but appeared to vary between “a few days” to six months. Five patients' treatment included K-wire fixation, 15 patients' did not. Of the 15 patients, six underwent nail removal, manipulation and splinting; nine (not stated explicitly, but by implication) underwent nail replacement, manipulation and splinting.	Case-series (level 4)	Clinical	K-wire: 1 (20%) osteomyelitis with eventual amputation 2 (40%) infections in the K-wire track Nail removal, manipulation, splinting: 3 (50%) nailbed infection 1 (17%) re-displacement at time of reduction – underwent K-wire fixation 3 (50%) re-displacement within a few days of reduction – one underwent K-wire fixation, for two the deformity was accepted (20° volar angulation at epiphysis of distal interphalangeal joint at 6 weeks, 10° at 6 months). Nail replacement, manipulation, splinting: 0 (0%) early or late re-displacement 9 (100%) normal or near-normal function at distal interphalangeal joint at 4–5 weeks.	No mention made of debridement nor the use of intraoperative or postoperative antibiotics.
Al-Qattan, 2001 [4], Saudi Arabia	23 patients, time to follow-up not disclosed. Five patients' treatment included K-wire fixation, 18 patients' did not.	Single-centre retrospective cohort study (level 2b)	Clinical	K-wire: 0 (0%) developed infection. No K-wire: 1 (6%) developed infection. K-wire: 0 (0%) developed residual flexion deformity. No K-wire: 3 (17%) developed residual flexion deformity of 10–15°.	Five patients were adults, with Seymour-like fractures. It is not clear what the spread of these five patients was within the two treatment groups. The study included two further adults who were discussed separately (total of 25 patients) and so could be excluded from this table.
Ganayem & Edelson, 2005 [5], Israel	7 patients, follow-up at 1–2.5 years, average 1.5 years. Six patients' treatment included K-wire fixation, one patient's did not.	Case-series (level 4)	Clinical	No patients developed infection. No patients developed deformity.	Small sample size.
Krusche-Mandl et al., 2013 [2], Austria	27 patients, 24 (89%) patients available for follow-up at 1–18 years, average 10 years. Five patients' treatment included K-wire fixation, 19 patients' did not.	Single-centre retrospective cohort study (level 2b)	Clinical	No patients developed infection. Nail dystrophy: K-wire: 1 (20%) No K-wire 5 (26%) Minor growth disturbance of distal phalanx and nail: K-wire: 2 (40%) No K-wire 3 (16%) Range of motion: All patients had a modified Kapandji index of 5/5 for extension. 23 patients had a modified Kapandji index of 5/5 for flexion, one had 0/5. Breakdown according to treatment not provided. No patients complained about pain. Visual analog scale score average 0.6, range 0–2. Breakdown according to treatment not provided. Patient satisfaction: 19 excellent, four good, one fair. Breakdown according to treatment not provided.	All cases of nail dystrophy and minor growth disturbance of distal phalanx and nail were associated with noteworthy luxation and nailfold laceration during primary assessment. The one case with modified Kapandji index of 0/5 for flexion had sustained a flexor digitorum profundus avulsion at time of injury. This same patient is the one that reported patient satisfaction of outcome as fair.
			Radiographic	Successful fracture healing in all patients, no malunion nor flexion deformities, no incomplete primary reduction. One patient had signs of a delayed union, with stable osseous union at 6 months. Treatment not provided. K-wire: 0 (0%) secondary displacement. No K-wire: 1 (5%) secondary displacement.	
Zhang et al., 2016 [6], China	26 patients, follow-up at 2–24 months, average 12 months. All patients' treatment included K-wire fixation.	Case-series (level 4)	Clinical	No patients developed infection. 1 (4%) nail deformity. 1 (4%) case where extension was restricted by 10°.	No comparison (i.e. treated without K-wire) group.
			Radiographic	All healed in 1–2 months. No non-union, malunion, re-displacement, nor premature epiphyseal closure.	
Lin et al., 2019 [7], United States of America	65 patients, follow-up at 0–333 days, median 30 days. Seven patients' initial treatment included K-wire fixation, 58 patients' initial treatment did not.	Case-series (level 4)	Clinical	6 (9%) superficial infections. Breakdown according to treatment not provided. 1 (2%) of these patients later developed an abscess and osteomyelitis. This patient was initially treated without K-wire. Unplanned operative intervention: K-wire: 0 (0%) No K-wire: 4 (7%) 3 (5%) patients required open reduction and K-wire due to unstable reduction or re-displaced fracture fragment. 1 (2%) patient did not receive incision and drainage in ED, so underwent later surgical exploration and debridement, open reduction, and K-wire fixation. She then developed an abscess and osteomyelitis which was treated with second operative incision and drainage, K-wire removal, and prolonged course of antibiotics. 24 patients had sufficient follow-up for documentation of nail regrowth. Breakdown according to treatment not provided. 1 (4%) nail dystrophy. This patient was initially treated without K-wire.	The 58 patients not initially treated with K-wire fixation were all treated in the ED, performed or supervised by a senior resident. At initial orientation, all residents receive detailed explanation of the management of Seymour fractures by an attending hand surgeon. Patients routinely follow-up within 1 week after treatment. If patients are doing well with no signs of complications, they are not routinely followed any further. The percentages for evidence of nail dystrophy, fracture healing, malunion, and physeal disturbance are not out of the total study population as radiographic and clinical follow-up were not available in some cases.
			Radiographic	47 patients received repeat X-rays on follow-up. Breakdown according to treatment not provided. 47 (100%) demonstrated radiographic evidence of fracture healing. 0 (0%) malunion. 2 (4%) physeal disturbance. These patients were treated without K-wire.	
Cha et al., 2021 [8], South Korea	12 patients, follow-up at least 2 years. All patients' treatment excluded K-wire fixation.	Case-series (level 4)	Clinical	1 (8%) superficial infection. Pain (visual analog scale) average 0.25. Disabilities of arm, shoulder, and hand score average 0.83. Active range of motion ratio average 99%.	No intervention (i.e. treated with K-wire) group. No statistically significant differences in pain, disability, active range of motion, dorsal angulation, nor length ratio when compared to contralateral side.
			Radiographic	No premature growth plate closures. Dorsal angulation average 0.50°. Length ratio average 98%.	
Perez-Lopez et al., 2021 [9], Spain	29 patients, follow-up at 2–36 months, average 11 months. 21 patients' treatment included K-wire fixation, eight patients' did not.	Single-centre retrospective cohort study (level 2b)	Clinical	Infection (all osteomyelitis): K-wire: 1 (5%) No K-wire: 4 (50%) Statistical difference. Functional range of motion: K-wire: 19 (90%) normal No K-wire: 6 (75%) normal No statistical difference. Physeal growth arrest: K-wire: 9 (43%) No K-wire: 1 (13%) No statistical difference.	No control between K-wire and no K-wire for other factors e.g. antibiotics, debridement, nailbed suture. Other results of the study included statistical significance in antibiotics relative to no antibiotics in having lower rates of infection.
Summary	206 patients, 75 patients' initial treatment included K-wire fixation, 131 patients' did not.		Complications	Infection: K-wire: 4 (5.3%) No K-wire: 10 (7.6%) Breakdown not provided: 5 Re-displacement of fracture: K-wire: 0 (0%) No K-wire: 8 (6.1%) Flexion deformity: K-wire: 1 (1.3%) No K-wire: 3 (2.3%) Nail dystrophy: K-wire: 2 (2.7%) No K-wire: 6 (4.6%) Minor growth disturbance of distal phalanx and nail: K-wire: 2 (2.7%) No K-wire: 3 (2.3%) Physeal disturbance: K-wire: 9 (12.0%) No K-wire: 3 (2.3%)	Due to the heterogeneity of the studies, no overall statistical analyses have been performed.

## 7 Discussion

Table 1 summarises 206 cases of Seymour fracture: 75 patients' treatment initially included K-wire fixation, 131 patients’ initial treatment did not. K-wire fixation appears to be associated with a higher rate of physeal disturbance but lower rates of infection, fracture re-displacement, and flexion deformity. However, due to heterogeneity of the studies, no overall statistical analyses have been performed. K-wire fixation may be associated with lower rates of infection due to its association with operative debridement and antibiotic treatment [10]. Internal fixation of the fracture by K-wires likely directly reduces the risk of fracture re-displacement and flexion deformity. Physeal disturbance may lead to growth disruption or arrest, producing deformities or impaired function. Ganayem & Edelson and Lin et al. cite Engber & Clancy when stating that physeal disturbance is thought to be caused by infection rather than direct injury to the growth plate [5,7,11]. However, the summary demonstrates a lower rate of infection and a higher rate of physeal disturbance with K-wires, suggesting that this assertion may need to be revised. Explanations for this finding may be that K-wire fixation is associated with more severe initial injuries, or that the process of K-wire fixation itself disrupts the physis, and that either may be additional contributory factors to physeal disturbance.

Long-term outcomes were favourable regardless of treatment modality (Table 1). Where measured, range of motion and patient satisfaction were all positive except for one patient who suffered flexor digitorum profundus avulsion at time of injury. Where measured, pain scores and disabilities of arm, should, and hand scores were low. There was successful fracture healing and good radiographic outcomes in most patients.

There are three main limitations to the above studies. First, five of the studies are case-series that do not make direct comparisons between those treated initially with K-wires and those who were not. Second, in the studies where such comparisons were made there was a paucity of statistical analyses. Finally, in the study where statistical analyses were performed, this was a single-centre retrospective cohort study that did not control for factors such as severity of initial injury, administration of antibiotics, wound debridement, nor nailbed suture, all of which may have influenced outcomes. Therefore, further research for Seymour fracture management is needed in the form of a randomised controlled trial, to confirm or refute the above findings. This will likely need to be multi-centre in view of the relatively infrequent presentation of Seymour fractures [10]. Such a study will likely involve debridement, open reduction, nailbed repair, nail plate fixation and the administration of antibiotics for all Seymour fractures. In those that demonstrate instability despite open reduction, all will require K-wire fixation. However, in those that do not demonstrate instability after open reduction, a position of equipoise could be argued (risk of physeal disturbance with K-wire, risk of re-displacement without, and unclear association of infection), and such patients be entered into the randomised controlled trial.

## 8 Clinical bottom line

Most patients will have normal clinical and radiographic findings at long-term follow-up regardless of treatment modality. K-wire fixation appears to be associated with a higher rate of physeal disturbance in children with a Seymour fracture. K-wire fixation appears to be associated with lower rates of infection, fracture re-displacement, and flexion deformity. There is a need for large-scale randomised controlled trials in Seymour fractures that do not demonstrate instability after open reduction to determine the treatment modality with better patient outcomes.

## Ethical approval

Ethical approval was not required for this study.

## Sources of funding

This research did not receive any specific grant from funding agencies in the public, commercial, or not-for-profit sectors.

## Author contributions

Riki Houlden devised the question, performed the literature search, appraised the papers, tabulated the results, and wrote the manuscript. He submitted and gave final approval of the version to be published.

## Registration of research studies

1. Name of the registry: not applicable
2. Unique Identifying number or registration ID: not applicable
3. Hyperlink to your specific registration (must be publicly accessible and will be checked): not applicable

## Guarantor

Riki Houlden.

## Consent

Not applicable.

## Declaration of competing interest

The author has no conflicts of interest to declare.
